# Supplementary material for: Development of PCR-Based Race-Specific Markers for Differentiation of Indian Fusarium oxysporum f. sp. cubense, the Causal Agent of Fusarium Wilt in Banana
Source: J Fungi (Basel). 2022 Jan 5;8(1):53. doi: 10.3390/jof8010053 (PMC8782045; doi:10.3390/jof8010053)

Supplementary Table S1. Details of reference *Foc* isolates (and their VCGs status) used in this study.

S. No.	*Foc* isolate number	VCG status of the isolate
1	24662	01213
2	Thai-7-24192	0125
3	W93/395 Aus	0124
4	W93/404	0125
5.	Mal 58	01217
6.	Thai-6-1	0124
7	Aus 23997	0128
8	Thai 6-1	0124
9	Taiwan 194	01213/16
10	W92/276	0129
11	Mal 42	01216
12	Mal 33	01218
13	Phil 8	0123
14	23746	0120
15	India 6	0124/5
16	India 1	0125
17	Phil 6	0126
18	RPSTNP	01212
19	24219	01220
20	Indo 20	0120
21	23631	01211
22	F9130	0121
23	23630	01210

Supplementary Table S2. List of primers designed specific to *Foc* races and their Gene ID.

S. No.	Gene ID	Forward primer	Reverse primer
***Foc* R1**			
1	XM_018396005.1	CTTACCAATTGGGGGCGTCT	GGTAGGTTGCGAACACCAGA
2	XM_018396005.1	TTGGGGCAAGGCATACAAGT	AGACGCCCCCAATTGGTAAG
3	XM_018396005.1	TTCCAATCAGCTTGGGGCAA	GAGACGCCCCCAATTGGTAA
4	XM_018394505.1	CTGAGCAGGAGGAACTCGAC	AAAGGCGGCTTCGGACATAA
5	XM_018394505.1	CCTCCGATACGGCTGATGAC	CCAACGTCTCGGTAACCAGT
6	XM_018394505.1	TACCTCCTTGGTCGACAGGT	CAGACTTCCAACGTCTCGGT
7	XM_018394754.1	GAATCTCTGCGCCTCGATGA	CGACAACATTAGAGCGCACG
8	XM_018394754.1	TGCGCCTCGATGACATTGTT	ACCGACAACATTAGAGCGCA
9	XM_018394754.1	ACTTCTGAATCTCTGCGCCTC	GGTGGGCCTTGGTCATAGAG
***Foc* R4**			
10	XM_031217723.1	GCCATGCTATTCCAACGATT	CATCCATTCTGCTGGGTTTT
11	XM_031217723.1	CAAAGCAGCTCTGACCATCA	GGCAAGGATACCGCAGAATA
12	XM_031217723.1	GCTATCCTGTGCACGATCCA	TGGTCAGAGCTGCTTTGGAG
13	XM_031196942.1	GAAGTCGACACCCACGAACT	AATTGTTGAACTGCAGCGGC
14	XM_031201004.1	GACTTCGTCACCCGCTACAA	CCGAGAACTGCCGGTAGATC
15	XM_031214609.1	AGAAGATCCTTACTGCCGCG	GTCACCGAACCAGTCTTCGT
16	XM_031214609.1	GACCGTCTGACCAATGATGC	TTTTCGAAGGTGGTCTGCAG
17	XM_031212605.1	TATCGGCATCTTCCAGTCGC	CGAGATCACCGTGGAGATGG
18	XM_031212605.1	AGGCCTGATCACGATTCCAA	CGCAGGTACAGAGTGTCGTA
19	XM_031212517.1	GTCGAAGGCCAGGAAGTGAA	GAGTGCGGATAGCCCTCTTC
20	XM_031216888.1	GATTTCCTCGACAACCAGGTG	TTCTGCACACCGTCGATGAT
21	XM_031212870.1	GAAATTCCGGAAAAGCGCGT	TTGAGCTCTTCCACCAGCTG
22	XM_031210754.1	AACATCCTGTCGCCGAAGTA	CGATGGTGTACTCGTGCTTG
23	XM_031209143.1	GGGTGAAGGGAGTGAAGCTC	AGCTGCATCAAACAAGCGTG
24	XM_031209143.1	GAAGTCGGCGTTCATGGATC	TCAAACAAGCGTGCCAATGA
25	XM_031203181.1	AACAACAACACCCCGTCCTC	TGTAGACGCTGCGCAGATAG
26	XM_031207491.1	ATCTCGAACAACAACACCCC	TCCTGCTCGCTGATGTAACC
27	XM_031214567.1	CGCGGCATCAACAAAGTCAT	CGGAATATCGTCGTCAGCGA
28	XM_031204365.1	AAGTCATCCTGGTCGGCAAT	CCGTAGTTGCCACCACCG
29	XM_031217424.1	CCAGGATGAGAAGACCCTGC	GGGAATTCACCGGTCTCGTT
30	XM_031198305.1	GGCATGTCTTCGCACTACAG	TGGAATCATGCGCATCACTG
31	XM_031213605.1	CAGGAGAGTCTTGCTTGCCA	AAACCCCCTTGACACTGGTG
32	XM_031213605.1	CGATCCATCTACCACTGCCT	CGGGCCGTTAAGTTTCGTAG
33	XM_031209245.1	GCGGATCTTCGTCGGGTAAT	CCAATGACTTCCTCGGCTGT
34	XM_031209245.1	CATCCAGGCTGCCAAGAATC	CTAAAGTGCCATATCCGCCG
35	XM_031209245.1	AGCCAATCAGAAGCTCTGCA	ATGCGAGAGATGTTGAGCCG
36	XM_031209245.1	TCTGCATTGCTATCTTCGGC	AATGCGAGAGATGTTGAGCC
37	XM_031197279.1	CCATCATTACCCGTCCCTCG	TCCAGCATGATCTTCTCCGC
38	XM_031216382.1	CTGTCACCCCATGTCGTACT	AGCATGATCTTCTCCGCCAG
39	XM_031211465.1	CCTGTTCAATGCCTTCGAGC	CTCACAACGCAGGATGGACT
40	KX434998.1 (SIX1)	TGATTTGCCGTGGAATGACA	TGGTCTTGACACGACCCA
***Foc* TR4**			
41	KF548063.1 (SIX8a)	CGCACTCTTACGTTGAGGAT	TCCACGCAACACTAGCTACT
***Foc* STR4**			
42	XM_031212766.1	CTTCGAGCTGACCATTTCCG	CAGCGAATCGAAGTACAGCG
43	XM_031208009.1	CCAAGAACAAACCGAAGCCG	GTTGGCAGTCATCCAGGTGA
44	XM_031197922.1	TCGCCAAGAACAAACCGAAG	TTTTGAAGCCCCACCAATCG
45	KX434398.1 (SIX7)	GCGCAAGTAGTCTTGCTTCC	ATTAAGCGGTTGGCGTATTG

Supplementary Figure S1. Forward and reverse primers binding site (A) and DNA sequence alignment of hypothetical protein (B) for *Foc* R1 of *Foc*R1F/*Foc*R1R.

A.

```
Results for linear 323 residue sequence "XM_018394505.1 Fusarium oxysporum f. sp. lycopersici 4287 hypothetical protein partial mRNA" starting "TACCTCCTTG"

          >>>F>>> 1 to 20
  1 TACCTCCTTGGTCGACAGGTAGCCGGTCAGTCCAGCGTTGAGGGCTACATCTCGGCTCTTATTCGAGGCTACCGCTGTGTCGAGATTGAC
  1          10        20        30        40        50        60        70        80
  1 ATGGAGGAACCAGCTGTCCATCGGCCAGTCAGGTCGCAACTCCCGATGTAGAGCCGAGAATAAGCTCCGATGGCGACACAGCTCTAACTG
 91 TGCTGGGATGGCAACTCTGGGCAGCCCGAGGTCAACCACGGTCACAGCCTTACGACCTCGATTAGTTTCCGTGAGGTCATAGCCACGATC
 91         100       110       120       130       140       150       160       170
 91 ACGACCCTACCGTTGAGACCCGTCGGGCTCCAGTTGGTGCCAGTGTCGGAATGCTGGAGCTAATCAAAGGCACTCCAGTATCGGTGCTAG
181 AATAAGTATGCCTTTGTTAAGTCGAGGTTCCCTCTCTGGATCCCCCTCGAAGTTCATTGCAACCCTAATCAGCAAGCCATCATGGTCAAC
181         190       200       210       220       230       240       250       260
181 TTATTCATACGGAAACAATTCAGCTCCAAGGGAGAGACCTAGGGGGAGCTTCAAGTAACGTTGGGATTAGTCGTTCGGTAGTACCAGTTG
                                  <<<R<<< 304 to 323
271 ATTATGAAGGAGGTCTTCGGCTCTCGACTGGTTACCGAGACGTTGGAAGTCTG
271         280       290       300       310       320
271 TAATACTTCCTCCAGAAGCCGAGAGCTGACCAATGGCTCTGCAACCTTCAGAC
```

B.

```
1. VCG0125 CM028857.1 Chr-15          TACCTCCTTGGTCGACAGGTAGCCGGTCAGTCCAGCGTCGAGGGCTACATCTCGGTTCTTATTCGAGGCTGCCGCTGTGTCGAG
2. VCG0124 CM027183.2 Chr-15          TACCTCCTTGGTCGACAGGTAGCCGGTCAGTCCAGCGTCGAGGGCTACATCTCGGTTCTTATTCGAGGCTGCCGCTGTGTCGAG
3. VCG0120 CM028827.1 Chr-15          TACCTCCTTGGTCGACAGGTAGCCGGTCAGTCCAGCGTCGAGGGCTACATCTCGGTTCTTATTCGAGGCTGCCGCTGTGTCGAG
4. VCGVCG01213/16 CM026318.2 Chr-15   TACCTCCTTGGTCGACAGGTAGCCGGTCAGTCCAGCGTCGAGGGCTACATCTCGGTTCTTATTCGAGGCTGCCGCTGTGTCGAG
5. VCG01220 CM028842.1 Chr-15         TACCTCCTTGGTCGACAGGTAGCCGGTCAGTCCAGCGTCGAGGGCTACATCTCGGTTCTTATTCGAGGCTGCCGCTGTGTCGAG
6. XM 018394505.1 Fol4287             TACCTCCTTGGTCGACAGGTAGCCGGTCAGTCCAGCGTCGAGGGCTACATCTCGGTTCTTATTCGAGGCTGCCGCTGTGTCGAG

1. VCG0125 CM028857.1 Chr-15          AACCCTAATCAGCAAGCCATCATGGTCAACATTATAAAGGAGGCCTTCGGCTCTCGACTGGTCACTGAGACGTTGGAAGCCTG
2. VCG0124 CM027183.2 Chr-15          AACCCTAATCAGCAAGCCATCATGGTCAACATTATAAAGGAGGCCTTCGGCTCTCGACTGGTCACTGAGACGTTGGAAGACTG
3. VCG0120 CM028827.1 Chr-15          AACCCTAATCAGCAAGCCATCATGGTCAACATTATAAAGGAGGCCTTCGGCTCTCGACTGGTCACTGAGACGTTGGAAGACTG
4. VCGVCG01213/16 CM026318.2 Chr-15   AACCCTAATCAGCAAGCCATCATGGTCAACATTATAAAGGAGGCCTTCGGCTCTCGACTGGTCACTGAGACGTTGGAAGACTG
5. VCG01220 CM028842.1 Chr-15         AACCCTAATCAGCAAGCCATCATGGTCAACATTATAAAGGAGGCCTTCGGCTCTCGACTGGTCACTGAGACGTTGGAAGACTG
6. XM 018394505.1 Fol4287             AACCCTAATCAGCAAGCCATCATGGTCAACATTATAAAGGAGGCCTTCGGCTCTCGACTGGTCACTGAGACGTTGGAAGACTG
```

Supplementary Figure S2. Forward and reverse primers binding site (A) and DNA sequence alignment of SIX8a (B) for *Foc* R4 of *Foc*R4F/*Foc*R4R.

A.

```
Results for linear 198 residue sequence "KF548063.1_Six8a_INDO 155" starting "CGCACTCTTA"

      >>>F>>> 1 to 20
    1 CGCACTCTTACGTTGAGGATGGACAGAACTAGGGGCGGAGGGACATCGAACGTGCTTGTCCTTTTGAGCGGGGGAGCGCTATCACATGCGGTACGGGAAGTAGTC
    1          10        20        30        40        50        60        70        80        90        100
    1 GCGTGAGAATGCAACTCCTACCTGTCTTGATCCCCGCCTCCCTGTAGCTTGCACGAACAGGAAAACTCGCCCCCTCGCGATAGTGTACGCCATGCCCTTCATCAG
                                                                             >>>R>>> 179 to 198
  106 CAGATCATGCCGGGTGAGGTGAAGAGCTTAGCTACTTTAGGTGCATCGACTGTCCAGTTTATAAACAATTGGTAGTAGCTAGTGTTGCGTGGA
  106     110       120       130       140       150       160       170       180       190
  106 GTCTAGTACGGCCCACTCCACTTCTCGAATCGATGAAATCCACGTAGCTGACAGGTCAAATATTTGTTAACCATCATCGATCACAACGCACCT
```

B.

1. CM026317.2 VCG01213/16 SIX8a	CGCACTCTTACGTTGAGGATGGACAGAACTAGGGGCGGAGGGACATCGAACGTGCTTGTCCTTTTGAGCGGGGGAGCGCTATCACA
2. CM028826.1 VCG0120 SIX8b	CGCACTCTTACGTTGAGGATGGACAAAACTAGGGGTGAACGGATATCGAACGTGCTTGTTATTTTGAGCGGGGGAGCGCTCTCACA
3. KF548063.1 Six8a INDO 155	CGCACTCTTACGTTGAGGATGGACAGAACTAGGGGCGGAGGGACATCGAACGTGCTTGTCCTTTTGAGCGGGGGAGCGCTATCACA
4. MN417222.1 Foc4 SIX8	CGCACTCTTACGTTGAGGATGGACAGAACTAGGGGCGGAGGGACATCGAACGTGCTTGTCCTTTTGAGCGGGGGAGCGCTATCACA
5. KF548064.1 Six8b Foc22615	CGCACTCTTACGTTGAGGATRGACAGAACTAGGGGCGGAGGGATATCGAACGTGCTTGTTATTTTGAGCGGGGGAGCGCTMTCACA

1. CM026317.2 VCG01213/16 SIX8a	CGGGTGAGGTGAAGAGCTTAGCTACTTTAGGTGCATCGACTGTCCAGTTTATAAACAATTGGTAGTAGCTAGTATTGCGTGGA
2. CM028826.1 VCG0120 SIX8b	CGGGTGCGGTGAGGAACTTAGCTACTTTAGGTGCATCGACTGTCCAGTTTCTACACAATTGGTAGTAGCTAGTATTGCGTGGA
3. KF548063.1 Six8a INDO 155	CGGGTGAGGTGAAGAGCTTAGCTACTTTAGGTGCATCGACTGTCCAGTTTATAAACAATTGGTAGTAGCTAGTGTTGCGTGGT
4. MN417222.1 Foc4 SIX8	CGGGTGAGGTGAAGAGCTTAGCTACTTTAGGTGCATCGACTGTCCAGTTTATAAACAATTGGTAGTAGATAGTGTTGCGTTGT
5. KF548064.1 Six8b Foc22615	CGGGTGAGGTGAGGAACTTGGCTACTTTAGGTGCATCGACTATCCAGTTTCTTACCAATTCCTACTAGATAGTGTTGCGTTGT

Supplementary Figure S3. Sequence comparison of SIX1a (A) and forward and reverse primers binding site (B).

A.

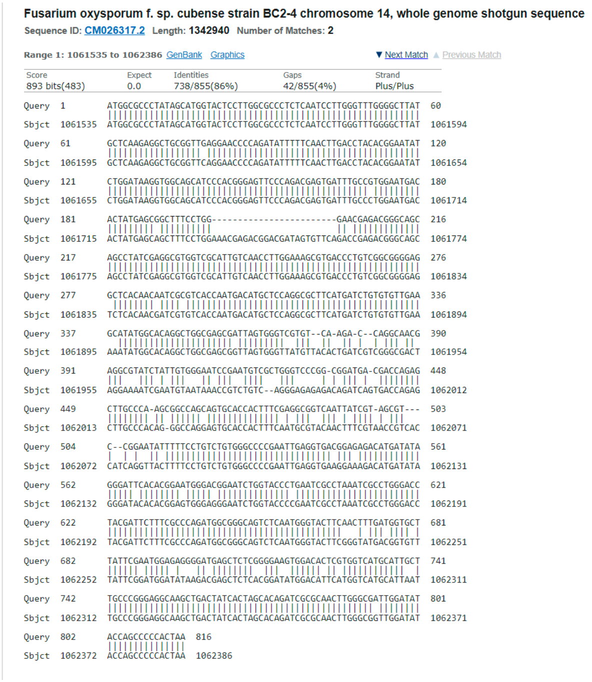

B.

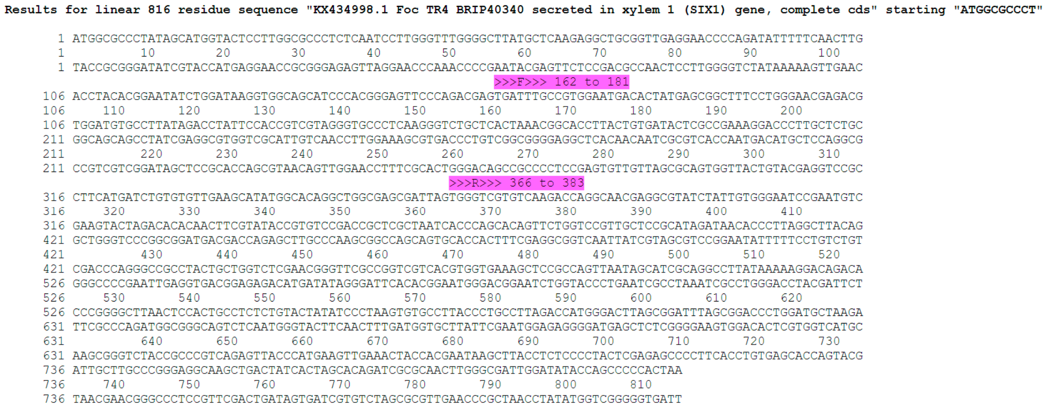

Supplementary Figure S4. Forward and reverse primers binding site (A) and multiple sequence alignment of SIX7a (B) for *Foc* STR4 of *Foc*STR4F/*Foc*STR4R.

A.

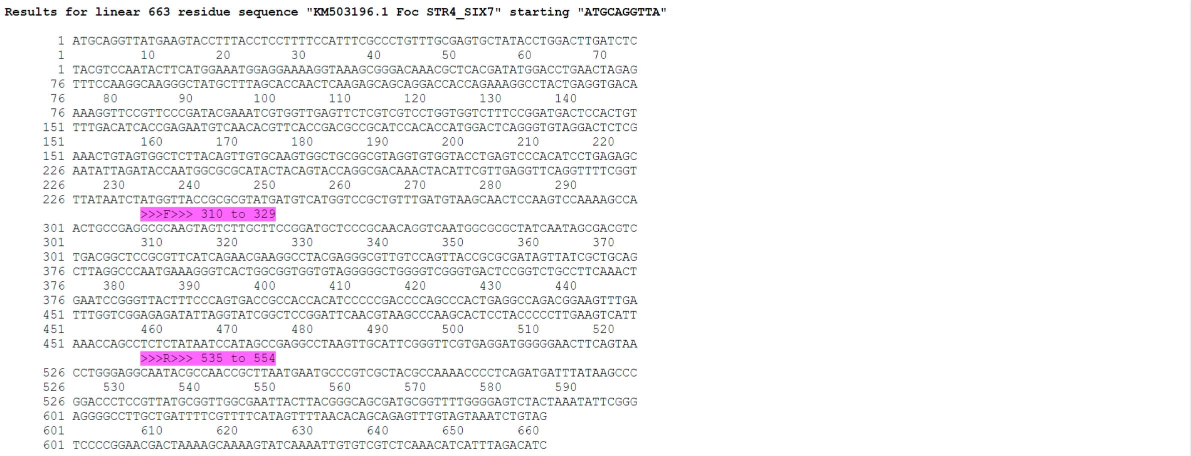

B.

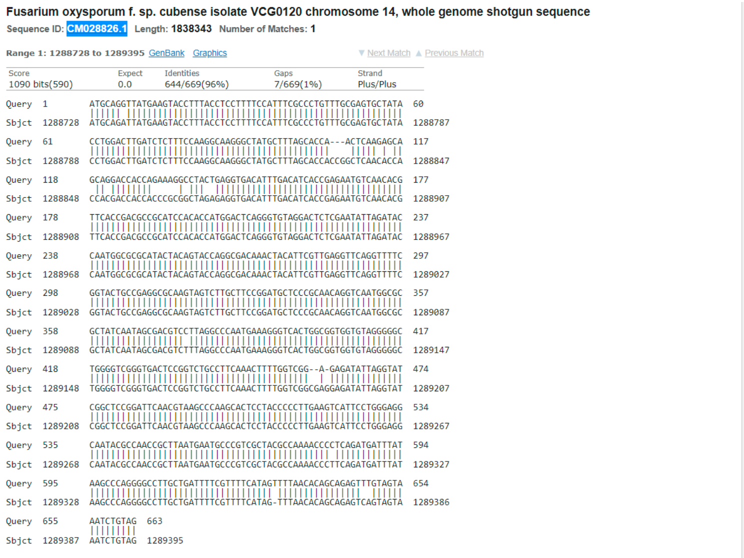

Supplement: Supplementary file 1 [file jof-08-00053-s001.zip › jof-1510505-SI.pdf]
